# Is nanomedicine still promising?

**DOI:** 10.18632/oncotarget.295

**Published:** 2011-06-11

**Authors:** Pubudu M. Peiris, Efstathios Karathanasis

**Affiliations:** Case Western Reserve University, Cleveland, Ohio

A recent study by Mann *et al.* [1] published in Oncotarget exploits the use of aptamers as a targeting ligand to develop site-specific delivery of liposomal nanocarriers. It has already been 17 years, since an article titled ‘Liposomes revisited’ was published co-authored by Lasic and Papahadjopoulos [2]. In essence, this publication marked the launch of the first success story of nanomedicine into the clinic and the beginning of an entire field. However, this was not an accident. The successful development of sterically stabilized liposomes was the product of 30 years of intensive research, since Bangham first discovered in the early 1960s that phospholipids in water form a vesicle enclosed in a bilayered lipid membrane [3, 4]. Not surprisingly, a pubmed search of the word ‘liposome’ resulted in 14,858 articles published between 1965 and 1995. The focus of these studies ranged from the *in vitro* particle stability to the effect of size, lipid composition and polymer coating of liposomes on their blood circulation, intratumoral accumulation and anticancer activity [5-7]. Furthermore, stable encapsulation of doxorubicin into the liposome with negligible leakage of the drug in blood circulation was achieved with the remote loading method against an ammonium sulfate gradient [8]. And finally voilá: a PEGylated unilamellar liposome composed of rigid phosphatidylcholine and cholesterol with a diameter of about 100 nm displayed prolonged circulation time and as a result an increased intratumoral accumulation and antitumor activity [6, 7].

So, what is the advantage of a long-circulating nanoparticle over conventional chemotherapies? It was shown that the therapeutic index of chemotherapeutics can be substantially improved, since nanoparticle delivery systems exploit a feature of tumor microenvironment, the so-called ‘Enhanced Permeability and Retention’ (EPR) effect [9]. While potent chemotherapeutic drugs are available to oncologists, the systemic dose of these agents is constrained by normal tissue tolerance, since these agents are distributed within cancer and healthy tissues in a non-specific manner. On the other hand, liposomes improve the safety profile of drugs due to their localization with high specificity in solid tumors while reducing off-target delivery [10, 11]. This unique feature has led to the development of various nanoparticle delivery systems, which are under preclinical and clinical evaluation [12]. The question that emerges then is: Could this old dog learn new tricks? This was promptly answered as it was recognized that the key feature of nanoparticles is the ability to combine more than one function by enabling the design of agents that target, image, and destroy tumors [13].

To further maximize the specificity towards cancer cells, targeting nanoparticles using a receptor-ligand system quickly attracted a lot of attention. Initially, nanoparticles were decorated with antibodies or small molecules that target receptors known to be over-expressed by tumors. Folate receptor-targeted liposomes were one of the first systems to be extensively studied. This is an ideal targeting system, since the folate receptor is an upregulated transmembrane receptor in various tumors [14]. While *in vitro* studies showed encouraging results proving that drugs delivered via folate receptor-targeted liposomes are rapidly transferred to the nuclei of target cells [15], the *in vivo* performance of these agents did not show an improved therapeutic efficacy in terms of prolonged survival [16]. Unfortunately, numerous studies have shown that the presence of folic acid or other targeting ligands (e.g. antibodies) on the surface of liposomes compromises their blood circulation time [15, 17, 18]. This is not unexpected as long circulation time of liposomal nanocarriers is due to a polyethylene glycol (PEG) shielding, and when targeting ligands are employed, they are usually conjugated on the distal end of the PEG chain resulting in recognition by the reticulo-endothelial system (RES) and accelerated clearance by the liver. Due to fact that nanoparticle extravasation is directly proportional to their concentration in blood [19], fewer targeted particles are able to reach the tumor site and consequently the gains from active targeting after extravasation into the tumor are compromised [20]. To address this major drawback, an elegant trick was proposed by McNeeley *et al.* [14]. A nanoparticle system targeting the folate receptor was developed based on a detachable PEG mask that ‘buries’ the targeting ligands when the nanoparticle is in circulation to maximize passive targeting to tumors. This detachable polymer coating can be removed by the administration of cysteine after nanoparticle extravasation to tumor is achieved to expose targeting ligands and promote active targeting only to cancer cells.

However, a simpler trick is to search for targeting moieties that are not immunogenic. Besides antibodies, many different types of affinity ligands, including humanized monoclonal antibodies, peptides and carbohydrate mimetics, have been developed [21, 22]. For example, oligopeptides identified by phase and bacterial display have lead to the development of targeted nanoparticles with comparable blood circulation as their non-targeted counterparts [23]. Aptamers based on oligonucleic acids present the most recent advancement in the search for high affinity and specificity ligands with improved pharmacokinetics and serum stability. Aptamers have been shown to mimic protein-binding molecules, and yet exhibit high binding affinity and selectivity [24]. For example, Pegaptanib is a vascular endothelial growth factor (VEGF)-specific aptamer that binds to human VEGF with K_D_ of 0.14 nM [25]. In contrast, affinity of VEGF neutralizing antibodies that block human VEGF binding to VEGFR-2 is about 0.4 nM [26]. Considering the affinity of peptides, a family of VEGF-binding peptides can bind to the VEGF receptor with K_D_ ranging from 0.14- 10 μM [27, 28]. Thus, the affinity of aptamers is very high and comparable to that of antibodies.

The study by Mann et al. [1] demonstrates the successful design of an active targeting liposomal system based on an aptamer ligand. They identified a thioated oligonucleotide aptamer (thio-aptamer) that binds E-selectin expressed on endothelial cells with high affinity and specificity. Liposomes decorated with the thio-aptamer on the outer surface exhibited (1) prolonged blood circulation that was comparable to the non-targeted formulation and (2) binding to tumor vasculature with high specificity. Thus, this work demonstrates that aptamers are an attractive alternative over antibody or peptide ligands based on their biological and chemical properties, such as small size, high affinity binding, selectivity, low immunogenicity, slow degradation kinetics, and a relatively low cost production process [24, 29, 30]. Aptamers are particularly well suited for nanotechnology applications, because they can be readily modified with functional groups that facilitate conjugation to nanomaterials [29].

In conclusion, studies like this show that nanomedicine has grown from an ‘exotic’ research area to a mainstream scientific field, which is governed by its own distinctive principles in terms of intravascular, transvascular and interstitial transport [31, 32]. Besides exploiting the multifunctionality of nanotechnology to develop targeting therapeutic, imaging and multi-component agents, the engineerability of nanoparticles has led to nanostructures with various shapes [33, 34] and new properties [35-37]. Therefore, it can be envisioned that the multifunctional and engineerable nature of nanotechnology can potentially facilitate agents that overcome the tumor biobarriers limiting the systemic delivery of active compounds.
